# Genetic Diversity, Chemical Components, and Property of Biomass *Paris polyphylla* var. *yunnanensis*

**DOI:** 10.3389/fbioe.2021.713860

**Published:** 2021-07-22

**Authors:** Nong Zhou, Lingfeng Xu, Sun-Min Park, Ming-Guo Ma, Sun-Eun Choi, Chuanling Si

**Affiliations:** ^1^Chongqing Engineering Laboratory of Green Planting and Deep Processing of Famous-Region Drug in the Three Gorges Reservoir Region, College of Biology and Food Engineering, Chongqing Three Gorges University, Chongqing, China; ^2^Department of Forest Biomaterials Engineering, College of Forest and Environmental Sciences, Kangwon National University, Chuncheon, South Korea; ^3^Beijing Key Laboratory of Lignocellulosic Chemistry, Research Center of Biomass Clean Utilization, Engineering Research Center of Forestry Biomass Materials and Bioenergy, College of Materials Science and Technology, Beijing Forestry University, Beijing, China; ^4^Tianjin Key Laboratory of Pulp and Paper, Tianjin University of Science and Technology, Tianjin, China

**Keywords:** *Paris polyphylla* var. *yunnanensis*, development, genetic diversity, molecular markers, chemical components

## Abstract

*Paris polyphylla* var. *yunnanensis* is a kind of biomass resource, which has important medicinal and economical values with a huge market. This review article aims to summarize the recent development of biomass *P. polyphylla* var. *yunnanensis*. The genetic diversity and chemical components of biomass *P. polyphylla* var. *yunnanensis* were reviewed based on the literature. Both the genetic diversity and genetic structure of biomass *P. polyphylla* var. *yunnanensis* were compared by using molecular marker technologies. All the extraction processes, harvest time, and drying methods on the chemical components were summarized in detail. The differences of arbuscular mycorrhizal fungi on the infection rate, diosgenin content, microorganisms, enzyme activities, rhizospheric environment, and endogenous hormones were discussed. This review article is beneficial for the applications of biomass *P. polyphylla* var. *yunnanensis* as a biomass resource in the biomedical field.

## Introduction

*Paris polyphylla* is a perennial herb and has various properties including clearing away heat, detoxification, detumescence, pain relief, and calming convulsion, which is widely used in the furuncle, carbuncle, sore throat, snakebite, tumbling pain, and convulsion fields. It was reported that *P. polyphyll*a is mainly distributed in the tropics of Europe and Asia (Liang and Victor, 2000). In China, it is mainly distributed in Sichuan Province, Guizhou Province, Yunnan Province, Guangxi Province, Guangdong Province, Jiangxi Province, Fujian Province, etc. *P. polyphylla* var. *yunnanensis* is located in Yunnan Province, Guizhou Province, Sichuan Province, and Chongqing city (Liu et al., 2015). *P. polyphylla* var. *yunnanensis* mainly contains chemical constituents of steroidal saponins, β-ecdysone, polysaccharide, flavone glycoside, and amino acids, which have important medicinal and economical values. Research shows that *P. polyphylla* var. *yunnanensis* has antibacterial and antitussive effects. In addition, the saponins of *P. polyphylla* var. *yunnanensis* have anticancer, analgesic, and sedative properties. The Chinese Pharmacopoeia (2015 edition) collected *P. polyphylla* var. *yunnanensis* as an important medicinal plant in 81 kinds of Chinese patent medicines, such as “Yunnan Baiyao,” “Gongxuening Capsule,” and so on (National Pharmacopoeia Commission, 2015).

With the rapid development of the Chinese traditional medicine industry, the demand for *P. polyphylla* var. *yunnanensis* as a raw material increased greatly. The wild *P. polyphylla* var. *yunnanensis* does not meet the requirements. More importantly, it takes 8–10 years from planting to harvesting and utilization of *P. polyphylla* var. *yunnanensis*. The price of *P. polyphylla* var. *yunnanensis* increased dramatically, resulting in the scarcity of the species. It was reported that more than 1,000 tons of *P. polyphylla* var. *yunnanensis* was utilized every year and about 80% of the wild *P. polyphylla* var. *yunnanensis* distributed in China has been utilized. Currently, wild resources cannot meet the demand of the market. The scarcity of *P. polyphylla* var. *yunnanensi* resources may become a bottleneck, restricting the sustainable development of the related pharmaceutical industry. Therefore, the development of artificial planting is an important choice to solve the shortage of *P. polyphylla* var. *yunnanensis* resources (Cunningham et al., 2018). In addition, the current research is about the total saponins of *P. polyphylla* var. *yunnanensis*. The basic research of *P. polyphylla* var. *yunnanensis* needs to be further explored in detail. It is necessary to separate and purify the active monomer components and study their activity to develop more valuable new drugs (Chen et al., 2016; Yang et al., 2019, 2021; Chen et al., 2020a; Ma et al., 2020; Wang et al., 2021). In the previous article, Negi et al. (2014) reviewed the developmental prospects of chemistry and biology of *P. polyphylla*. After that, Cunningham et al. (2018) assessed the scale of *P. polyphylla* trade, reviewing evidence about the impacts of wild harvest on *P. polyphylla* populations and the role of cultivation as an alternative to wild harvest. More recently, Pei et al. (2020) reviewed medicinal records of ethnic minorities for *P. polyphylla* in China and indicated the applications of authentic evaluation techniques of ethnobotanical medicinal plant of genus *P. polyphylla*.

The purpose of this review article is to introduce the development and problem of *P. polyphylla* var. *yunnanensis* based on previous research. The article analyzed the genetic diversity and structure of wild populations and cultivated populations of *P. polyphylla* var. *yunnanensis* and compared the different molecular markers methods. Then, the effects of fungi on the properties of *P. polyphylla* var. *yunnanensis* were also reviewed. Finally, the problems and suggestions were given on the *P. polyphylla* var. *yunnanensis* based on our present knowledge. It was expected that this review article favored the applications of *P. polyphylla* var. *yunnanensis* in the natural medicine field. More research should be focused on the artificial planting, genetic diversity, structure, properties, and applications of *P. polyphylla* var. *yunnanensis*.

## The Population Genetics of *Paris polyphylla* var. *yunnanensis*

In the literature, *P. polyphylla* var. *yunnanensis* was first recorded in Shennong’s Herbal Classic of Materia Medica under the name of “Zaoxiu.” After that, it was recorded in famous books, such as “Supplementary Records of Famous Physicians,” “Tang Materia Medica,” “Rihuazi Materia Medica,” and so on, containing the contents of the morphology, growth environment, and properties of *P. polyphylla* var. *yunnanensis*.

There are many reports on the genetic diversity and genetic structure of *P. polyphylla* and its related species inside and outside the region (He et al., 2007; Yang et al., 2015; Zhao X.P. et al., 2020). A random amplified polymorphic DNA (RAPD) molecular marker was developed by Wiliam and Welsh in 1990 to detect DNA polymorphism by PCR. This research indicated the important applications of *P. polyphylla*. For example, Zhang et al. (2004) reported the genetic diversity of *P. polyphylla* var. *yunnanensis* from Yunnan, Guangxi, and Vietnam using RAPD molecular marker.

Simple sequence repeats (SSRs) molecular marker is a DNA molecular marker technology based on PCR. In He et al. (2007), the genetic diversity and genetic structure of cultivated and wild *P. polyphylla* were reported based on inter-simple sequence repeat (ISSR) molecular marker. It showed that the genetic diversity (polymorphism) of the cultivated population was higher than that of the wild population (0.153 vs. 0.151). The result indicated that the artificial planting was a realistic strategy for solving the shortage of *P. polyphylla* var. *yunnanensis* resource. In addition, the genetic diversity (polymorphism) of 62 individuals of *P. polyphylla* var. *yunnanensis* from Yunnan Province was explored based on SSRs molecular marker (Song et al., 2015). They developed 10 pairs of primers to provide a primer basis for the SSR marker of *P. polyphylla* var. *yunnanensis*. In Chen et al. (2017), the genetic diversity of 115 samples from 5 populations in Yunnan was analyzed based on simple sequence repeats (SSR) molecular marker. The results showed that the expected heterozygosity was 0.774 at the species level and 0.655 at the population level, respectively, indicating the high genetic diversity of *P. polyphylla* var. *yunnanensis*.

Amplified fragment length polymorphism (AFLP) molecular marker was a method for detecting DNA polymorphism developed by Vos et al. (1995) in Netherlands in 1995, which is a combination of RFLP and PCR. Li et al. (2018) used AFLP molecular marker to analyze the genetic diversity of *P. polyphylla* var. *yunnanensis* from 15 populations in Yunnan Province. Research group investigated the genetic diversity (polymorphism) and genetic structure of *P. polyphylla* var. *yunnanensis*, 15 wild populations, and 17 cultivated populations, based on AFLP molecular marker and cpDNA fragments (Huang Y. et al., 2019; Hu et al., 2014, 2016). According to the analysis of molecular variance (AMOVA), only 1.35% of genetic variation existed between wild and cultivated populations, indicating that there was no obvious genetic differentiation between wild and cultivated populations, due to the relatively short history of the domestication of cultivated populations. The chloroplast gene, cpDNA fragment (trnL TRNF), was used to analyze the phylogeography of wild *P. polyphylla* var. *yunnanensis* (Zhao J.J. et al., 2020). The genetic structure of 17 cultivated populations showed mixed provenances in the cultivation base, leading to unstable quality. The cpDNA fragment (trnL TRNF) is a rapid, economic, and effective strategy to analyze the phylogeography of *P. polyphylla* var. *yunnanensis*.

A variety of molecular marker technologies have been developed rapidly, which were widely used in plant genetic research (Chen et al., 2020b; Liu H. et al., 2021a,b; Liu K. et al., 2021; Liu et al., 2021). Molecular marker technologies is used in the applications of the breeding and production of animals and plants, which mainly focus on gene mapping, assisted breeding, disease treatment, etc. The development of molecular marker technology is a hot topic in the field of molecular biology (An et al., 2019; Du et al., 2019, 2021; Li et al., 2019; Xu J. et al., 2021). With the rapid development of molecular biology theory, molecular marker technology will be developed with fast analysis speed, low cost, and more information (Ma et al., 2021a,b; Xu et al., 2020; Xu R. et al., 2021).

Yang et al. (2016) reported the bacterial strain, py1294(T), isolated from a root of *P. polyphylla* var. *yunnanensis* by using a polyphasic approach to clarify its taxonomic position. They indicated that the strain py1294(T) formed a well-supported clade with *Oceanobacillus damuensis* PT-20(T) (97.9% sequence similarity) within the genus *Oceanobacillus*. It obtained the DNA-DNA relatedness value of 29.7% between strain py129 (T) and *O. damuensis* PT-20(T). Jia et al. (2016) isolated two actinobacteria, designated strains NEAU-JM1(T) and NEAU-CL2(T), from volcanic sediment and the rhizosphere soil of *P. polyphylla*. They established the taxonomic positions of these strains and concluded these two species of the genus Catellatospora.

As early as Xuan et al. (2010) isolated fungal endophyte from *P. polyphylla Smith* var. *yunnanensis* and identified its antibacterial ability. Authors obtained 18 fungal endophytes, parasitizing the famous Chinese medicinal plant *P. polyphylla Smith* var. *yunnanensis*, and evaluating the effect of endophytes on the growth of human pathogenic microbes *in vitro* using disk diffusion assay. Then, Zhou et al. (2012) investigated the effects of fungal elicitors on the secondary metabolite steroidal saponin in *P. polyphylla* var. *yunnanensis*. There was selectivity between arbuscular mycorrhizal fungal and *P. polyphylla* var. *yunnanensis*, and Glomus intraradice was the most appropriate strain for inoculation of *P. polyphylla* var. *yunnanensis*. Recently, Wang Y. et al. (2020) investigated the effects of altitude on the community composition of endophytic fungal communities and the differentiation of endophytic microorganisms among different niches in *P. polyphylla*. The author also indicated that the structural variability in the rhizosphere fungal community was significantly lower than that in the endophytic communities, confirming the presence of niche differentiation among members of the endophytic microbial community.

There is a connection between the genetic and the fungal sections; however, there are few reports on this topic. Therefore, more rapid attention should be paid on the connection between the genetic and the fungal sections of *P. polyphylla* var. *yunnanensis* in the near future.

## The Chemical Components of *Paris polyphylla* var. *yunnanensis*

The polysaccharides have a variety of pharmacological and biological functions, such as antioxidant, antitumor, antiviral, immune regulation, and anti-inflammatory (Si et al., 2008a,b, 2009, 2013; Dai et al., 2020a,b). *P. polyphylla* var. *yunnanensis*, as an important plant polysaccharide, has attracted more attention in recent years (Jing et al., 2017; Shen et al., 2018). *P. polyphylla* has been reported to contain a variety of chemical components including daucosterol, polyphyllin D, beta-ecdysterone, Paris saponins I, II, V, VI, VII, H, dioscin, oligosaccharides, heptasaccharide, octasaccharide, trigofoenoside A, protogracillin, Paris yunnanosides G-J, padelaoside B, and pinnatasterone, which have biological properties, such as anticancerous, antitumor, cytotoxic, anthelmintic, antimicrobial, antiangiogenic, immunostimulating, contractile, and hemostatic (Negi et al., 2014; Liu et al., 2020a,b; Wang H. et al., 2020). There are many reports on the extraction and applications of polysaccharides, *P. polyphylla* var. *yunnanensis* (Huang et al., 2007; Chan et al., 2011; Wu et al., 2012; Qin et al., 2016).

As early as Zhou et al. (2003) investigated the heptasaccharide and octasaccharide isolated from *P. polyphylla* var. *yunnanensis* and their plant growth-regulatory activity. In Shen et al. (2014) reported the optimization of the extraction process and antioxidant activity of polysaccharides from the leaves of *P. polyphylla.* The monosaccharide components of *P. polyphylla* var. *yunnanensis* were obtained using the hydrolysis method at 110°C temperature for 6 h in 2 mol⋅L^–1^ trifluoroacetic acid (Wang et al., 2019). The recoveries of five monosaccharide components were 92.38–99.98%. The polysaccharides of *P. polyphylla* var. *yunnanensis* were mainly composed of glucose, mannose, galactose, rhamnose, and arabinose, whose molar ratio in wild species was 219.22:28.23:1.83:1.44:1.00.

Research group extracted the polysaccharide of *P. polyphylla* var. *yunnanensis* through three different extraction processes, such as reflux extraction, hot water extraction, and ultrasonic-assisted extraction (Zhou et al., 2014). It was found that the *P. polyphylla* var. *yunnanensis* extracted by ultrasonic-assisted extraction method had the highest polysaccharide content of 24.32 mg g^–1^. We also found the change content of diosgenin significantly in *P. polyphylla* var. *yunnanensis* at different harvest times (Zhou et al., 2010). It has been observed that the content was increased gradually from April to June, the highest content was from May to June, and the content was decreased sharply from June to July. The influence of the drying method on the diosgenin of *P. polyphylla* var. *yunnanensis* was also explored (Zhou et al., 2015b). It was found that the color changed from white to light brown and powdery using the drying at 35C, natural shade drying, and sun-drying had good color, while the color changed from brown to dark brown and horny by those of other drying methods. Different drying methods had different effects on the diosgenin of *P. polyphylla* var. *yunnanensis.*

As early as Lee et al. (2005) investigated the effects of polyphyllin D, a steroidal saponin in *P. polyphylla*, on growth inhibition of human breast cancer cells and in xenograft. Then, Yan et al. (2009) confirmed anticancer activity of steroid saponins of *P. polyphylla* var. *yunnanensis* by both *in vitro* and *in vivo.* After that, Wang et al. (2010) investigated the anthelmintic activity of crude extracts and pure compounds from the rhizomes of *P. polyphylla*. In addition, steroidal saponins from the rhizome of *P. polyphylla* and their cytotoxic activities were also researched using two new furostanol saponins and one new spirostanol saponin isolated from the rhizome of *P. polyphylla* var. *yunnanensis*, together with 18 known steroidal saponins (Zhao et al., 2009; Hu et al., 2017, 2018). There was a report on the steroidal saponins with antimicrobial activity from stems and leaves of *P. polyphylla* var. *yunnanensis* (Qin et al., 2012). The effects of nine different drying methods on the content of total saponins in rhizomes of *P. polyphylla* var. *yunnanensis* and the differences in antioxidant activity were also studied (Zhang et al., 2016). The total saponin content of different drying methods was significantly different in rhizomes of *P. polyphylla* var. *yunnanensis*.

Four saponins were investigated in *P. polyphylla* var. *yunnanensis* with different growth years (Zhang et al., 2011). It was found that the content of saponins of *P. polyphylla* var. *yunnanensis* in 3-year-old herbs was lower than those in 4-year-old and 5-year-old herbs. The contents of nine steroidal saponins were reported in rhizomes and fibrous roots of *P. polyphylla* var. *yunnanensis* (Gu et al., 2020a). The quite different content of steroidal saponins in 32 samples of *P. polyphylla* var. *yunnanensis* was obtained from different areas. Most of them could detect nine kinds of steroidal saponins in rhizomes. The *P. polyphylla* var. *yunnanensis* had rich contents of saponins I and II and relatively low contents of saponins VI and dioscin. The effects of ultrasonic extraction and hot water extraction on the extraction of nucleoside from *P. polyphylla* var. *yunnanensis* were analyzed using rhizome as experimental materials and the total extraction amount of six nucleosides (cytidine, uridine, guanosine, thymidine, adenosine, and deoxyadenosine) as the index was analyzed, in which the extraction amount was determined by high-performance liquid chromatography (HPLC) (Yang et al., 2017). The results showed that the total extraction amount of six nucleosides from rhizomes of *P. polyphylla* var. *yunnanensis* by ultrasonic extraction was higher than that of the hot water extraction method. The total extraction amount of cytidine, uridine, guanosine, thymidine, adenosine, and deoxyadenosine from rhizomes of *P. polyphylla* var. *yunnanensis* was 1.422 mg g^–1^, which was higher than the theoretical value of 1.343 mg g^–1^.

The amino acids of *P. polyphylla* var. *yunnanensis* were investigated in 27 samples of *P. polyphylla* var. *yunnanensis* collected from Yunnan Province, Guizhou Province, and Sichuan Province (Gu et al., 2020b). The good linear relationship of 15 amino acids in fibrous roots of *P. polyphylla* var. *yunnanensis* was observed, among which the highest contents of aspartic acid and glutamic acid were found in all producing areas. The transplanting *P. polyphylla* var. *yunnanensis* had high contents of amino acids in fibrous roots compared with that of the wild *P. polyphylla* var. *yunnanensis.* The contents of inorganic elements were investigated in both Yunnan Province and Guizhou Province (Yang et al., 2018). The good linear relationship of 14 inorganic elements and the average recovery rate of 90.81 –109.73% was found. There are obvious differences among the mass fraction and composition structure ratio of 14 inorganic elements in *P. polyphylla* var. *yunnanensis* medicinal material from different places.

Based on the previous reports, it was found that the extraction processes, harvest time, drying methods, growth years, extraction methods, and other factors had an impact on the chemical components of *P. polyphylla* var. *yunnanensis*. More research should be developed on the chemical components of *P. polyphylla* var. *yunnanensis* in detail.

## The Effects of Fungi on the Content and Properties of *Paris Polyphylla* var. *Yunnanensis*

In the literature, the fungi were reported to play an important role in the contents and properties of *P. polyphylla* var. *yunnanensis* (Li et al., 2008; Huang et al., 2009; Liu et al., 2017). The effects of 28 arbuscular mycorrhizal fungi on the infection rate and diosgenin content of *P. polyphylla* var. *yunnanensis* were explored in sterilized soil (Wang et al., 2018). The results indicated that there was a good symbiotic relationship between *P. polyphylla* var. *yunnanensis* and arbuscular mycorrhizal fungi, and the significant increase in infection rate. More importantly, it was found that there was no significant difference between the diosgenin contents of *P. polyphylla* var. *yunnanensis* and the control group; however, it was observed a significant increase in diosgenin content of 41.40 mg g^–1^ after treatment with Svi fungi. The effects of inoculation periods on growth and steroidal saponin content of *P. polyphylla* var. *yunnanensis* seedlings infected by arbuscular mycorrhizal fungi were investigated (Huang Y.P. et al., 2019). The strong infection rate of arbuscular mycorrhizal fungi in different inoculation periods, the increased protective enzyme activity, photosynthetic pigment, and soluble sugar content were found. The inoculation time had a certain influence on the growth and development of *P. polyphylla* var. *yunnanensis* seedlings infected by arbuscular mycorrhizal fungi and the saponins content of *P. polyphylla* var. *yunnanensis*. The effects of arbuscular mycorrhizal fungi on the number of microorganisms and enzyme activities in rhizosphere soil of *P. polyphylla* var. *yunnanensis* were investigated (Ou et al., 2016). One can observe that there was a certain degree of mutual selectivity between *P. polyphylla* var. *yunnanensis* and arbuscular mycorrhizal fungi. After the induction of arbuscular mycorrhizal fungi, the different arbuscular mycorrhizal fungus had different effects on the number of microorganisms, microbial biomass carbon, and soil enzyme activities in rhizosphere soil of *P. polyphylla* var. *yunnanensis*.

Research group (Zhou et al., 2020) studied the effect of arbuscular mycorrhizal fungi treatments of different combinations on the rhizospheric environment of *P. polyphylla* var. *yunnanensis*. The inoculation of arbuscular mycorrhizal fungi could regulate the spore densities and the colonizations of *P. polyphylla* var. *yunnanensis* rhizosphere arbuscular mycorrhizal to improve the root activity. Effects of arbuscular mycorrhizal fungi on endogenous hormones in *P. polyphylla* var. *yunnanensis* were reported (Zhou et al., 2017). It was found that the inoculation of exogenous arbuscular mycorrhizal fungi could improve the mycorrhizal infection rate and seedling rate of *P. polyphylla* var. *yunnanensis* seedlings. The arbuscular mycorrhizal fungi had different rules on the changes in the rhizome and fibrous root of *P. polyphylla* var. *yunnanensis* seedlings. Fifty strains of endophytic fungus were isolated from phloem, xylem, and seed of rhizome of *P. polyphylla* var. *yunnanensis* collected from Yunnan Province (Zhou et al., 2004). The most endophytic fungi was isolated from the phloem of the rhizome. In addition, steroids in the fermentation cultures of 32 strains of endophytic fungus was detected, and the yield of steroids in the fermentation cultures of five strains of endophytic fungus exceeded 50 mg L^–1^.

Zhang et al. (2019) reported the arbuscular mycorrhizal fungi suitable for the growth and development of *P. polyphylla* var. *yunnanensis* seedlings screened with mycorrhizal viability, rhizome biomass, and active components as indexes. It was found that the 28 arbuscular mycorrhizal fungi inoculated with *P. polyphylla* var. *yunnanensis* formed a symbiotic system, and could improve the mycorrhizal viability of *P. polyphylla* var. *yunnanensis* seedlings. Research group found that exogenous arbuscular mycorrhizal fungi could regulate the spore density, infection rate, and infection intensity of arbuscular mycorrhizal fungi in roots of *P. polyphylla* var. *yunnanensis*, and enhance the activities of succinate dehydrogenase and alkaline phosphatase of hyphae in roots of *P. polyphylla* var. *yunnanensis* (Zhou et al., 2015a). After treated with arbuscular mycorrhizal fungal elicitors, the different effects of different arbuscular mycorrhizal fungus on different steroidal saponins in rhizomes of *P. polyphylla* var. *yunnanensis* was observed. The application of arbuscular mycorrhizal fungi could improve the medicinal quality of *P. polyphylla* var. *yunnanensis*.

Indeed, the mechanism at the molecular level is also needed to be further studied in detail, which is important for the applications of *P. polyphylla* var. *yunnanensis*. In addition, the utilization of biotechnological processes should be developed for the production of *P. polyphylla* var. *yunnanensis* under controlled conditions.

## Conclusion and Perspectives

There are many reports on the genetic diversity, genetic structure, and chemical components of *P. polyphylla* var. *yunnanensis*. The fungi also affected the contents and properties of *P. polyphylla* var. *yunnanensis;* however, there are few review articles on the *P. polyphylla* var. *yunnanensis*. This review article provided the recent development about the genetic diversity and structure of *P. polyphylla* var. *yunnanensis* based on the literature. More attention should be paid to the genetic diversity and genetic structure, which favored the replacement of the wild population by the cultivated population. In addition, the differences between wild population and cultivated population should be investigated in detail, which is important for the applications of *P. polyphylla* var. *yunnanensis.* Especially, the effect of areas on the quality and chemical components of *P. polyphylla* var. *yunnanensis* should be explored. Furthermore, more methods should be developed on the extraction and purity of the chemical components of *P. polyphylla* var. *yunnanensis.* The mechanism between chemical components and biological activities should be discussed by more experimental results. Furthermore, the utilization of fungi on the properties of *P. polyphylla* var. *yunnanensis* should be developed in detail. More biomedical properties should be investigated. It is well-known that *P. polyphylla* var. *yunnanensis* is an important biomass resource in the natural medicine field (Liu H. et al., 2021a,b). Therefore, the mechanism between chemical components and properties should be researched in depth. We expected that this review article is beneficial to expand the applications of *P. polyphylla* var. *yunnanensis* in the biomedical field.

## Author Contributions

NZ, LX, and M-GM: investigation. S-MP, S-EC, and CS: supervision. NZ and M-GM: writing – original draft. M-GM, S-MP, LX, S-EC, and CS: writing – review and editing. All authors contributed to the article and approved the submitted version.

## Conflict of Interest

The authors declare that the research was conducted in the absence of any commercial or financial relationships that could be construed as a potential conflict of interest.
